# Editorial: Utilizing omics strategies to discover new drug targets for cancers

**DOI:** 10.3389/fphar.2024.1526976

**Published:** 2024-11-27

**Authors:** Shujun Zhang, Chen Xue, Xinyu Gu

**Affiliations:** ^1^ Department of Infectious Diseases, The First Affiliated Hospital, College of Clinical Medicine, Henan University of Science and Technology, Luoyang, Henan, China; ^2^ State Key Laboratory for Diagnosis and Treatment of Infectious Diseases, National Clinical Research Center for Infectious Diseases, National Medical Center for Infectious Diseases, Collaborative Innovation Center for Diagnosis and Treatment of Infectious Diseases, The First Affiliated Hospital, Zhejiang University School of Medicine, Hangzhou, Zhejiang, China; ^3^ Department of Oncology, The First Affiliated Hospital, College of Clinical Medicine, Henan University of Science and Technology, Luoyang, Henan, China

**Keywords:** omics, new drugs, cancer, precision medicine, drug targets

Cancer is a major public health issue and a significant contributor to the global disease burden. Since 2010, different kinds of cancer have become the main cause of deaths, with the incidence, mortality and disease burden all escalating. Data shows that approximately 10 million people die from cancer globally each year (Qi et al., 2023). The incidence and mortality rates of cancer increase exponentially with age, and given the aging world population, it is expected that the number of cancer-related deaths worldwide will continue to rise, causing huge public health costs. Currently, the main treatment methods for cancer include surgery, radiotherapy, chemotherapy, and targeted therapy. Surgery is usually the first-line approach for most tumors, that is suitable for patients in the early stage. Radiotherapy and chemotherapy are generally used as complementary options after surgery or for cancer patients who have no possibility of surgery. Targeted therapy addresses gene mutations and has better efficacy, while individual differences and the emergence of drug resistance necessitate discovering new cancer drug targets and the development of more targeted therapeutic drugs.

The pathogenesis of cancer involves complex reorganizations of various genetic, transcriptional, proteomic, and metabolomic processes that drive tumor development. Several omics technologies have been shown to exhibit great potential in cancer research, which include genomics, epigenetics, transcriptomics, proteomics, and metabolomics. Genomics is one of the essential omics technologies in this field. Genome sequencing enables researchers to identify gene mutations that drive cancer progression. Meanwhile, epigenetics analysis enables a comprehensive description of the epigenetic profile of cancer patients, referring to the occurrence, growth, metastasis, and immune evasion of tumors. Transcriptomics analysis can capture changes in and differences between gene expression patterns between cancer cells and normal cells, providing a comprehensive perspective on the molecular changes that occur in cancer. Proteomics can identify and quantify proteins present in tissues or cells, providing insights into the functional changes that occur in cancer. Metabolomics analysis can detect alterations in the metabolic profile in cancer, thereby providing a deeper understanding of the metabolic dependences that drive tumor growth.

More specifically, genomics examines DNA sequences and deciphers the genetic information encoded in the genome. By comparing the genomes of tumor cells with those of healthy cells, scientists can pinpoint the specific genetic mutations that drive tumor growth. These findings provide clues for identifying potential drug targets that can be used to develop precise targeted therapies. Epigenetic changes can affect gene expression and function through the chemical modifications of nucleotides and proteins. There is growing evidence that epigenetic changes play an important role in the occurrence and development of human cancers; many epigenetic biomarkers have also been found to be targets for cancer therapy. Another important omics strategy is transcriptomics, which involves studying the expression of genes in tissues or cells. By identifying genes that are over- or underexpressed in cancer, researchers can prioritize these as candidates for targeted drug research. Proteomics also facilitates the discovery of new drug targets, while identifying proteins that are dysregulated in cancer support the development of targeted therapies. Restoring normal protein function or inhibiting abnormal protein activity can correct abnormal cell states and mitigate disease progression. Metabolomics is the study of small molecules in metabolic pathways, playing an important role in the discovery of new drug targets for cancer therapy. Identifying specific metabolic pathways critical to cancer cell survival opens up new avenues to develop drugs that can selectively target these pathways.

SCLC is an aggressive neuroendocrine (NE) tumor with strong proliferation and metastasis potential, significant drug resistance, and poor prognosis (Megyesfalvi et al., 2023). Although targeted therapy and immunotherapy have greatly improved the prognosis of non-SCLC patients (NSCLC), the advancement of SCLC treatments has been slow, with no significant improvement achieved in the survival rate of patients, therefore these are still outside the field of precision medicine. By integrating mRNA, protein and phosphorylation data from 107 SCLC tumors, unsupervised clustering based on non-negative matrix decomposition (NMF) was applied to divide SCLC into four subtypes: NMF1, NMF2, NMF3 and NMF4 (Liu et al., 2024). Firstly, multi-omics analysis revealed that nmf1 subtypes were mainly enriched in cell cycle, DNA damage, chromatin organization, and epigenetic regulatory pathways, and had a strong response score to ATR and TOP1 inhibition. The level of NOTCH ligand delta-like protein 3 (DLL3) was highest in the nmf2 subtype. Therefore, this subtype is likely to benefit from therapies targeting DLL3. Secondly, phosphorylated proteomic data showed that RTK signaling activity was significantly upregulated in the nmf3 subtype. Thus, targeting RTK may be a potential strategy to treat this subtype. The nmf4 subtype is characterized by high MYC expression and enrichment of RNA metabolic pathways, and is preferentially associated with AURKA amplification, further suggesting potential opportunities to target AURKA. Multiomics analysis in SCLC can expand our understanding of the molecular events of these aggressive malignancies and contribute to more effective clinical treatments for this cancer type. In triple negative breast cancer (TNBC), genomic and transcriptomic strategies have indicated that programmed cell death ligand-1 (PD-L1) has high mutational activity and is overexpressed in about 20% of TNBC patients, thus may serve as a potential therapeutic target (Kudelova et al., 2022). Upon further study, the anti-PD-L1 antibody atezolizumab became the first FDA-approved immunotherapy drug for locally advanced or metastatic TNBC. In addition, the application of omics analysis has significantly facilitated deriving targeted therapies of other cancers, such as breast cancer (Neagu et al., 2023), lung cancer (Yan et al., 2024), gastric cancer (Hou et al., 2023), hematological malignancies (Rosenquist et al., 2023), etc. The integration of these omics approaches has greatly accelerated the discovery of new drug targets for cancer therapy.

Overall, omics-based research strategies have great potential in cancer research, deepening our understanding of tumor behavior considerably and providing a comprehensive cancer map to guide tumor precision therapies. A variety of available omics techniques can help scientists to extensively study the molecular characteristics of tumors and identify potential drug targets, revolutionizing the field of cancer research.
